# Dosimetric impact of density variations in Solid Water 457 water‐equivalent slabs

**DOI:** 10.1120/jacmp.v12i3.3398

**Published:** 2011-04-22

**Authors:** Dale W. Litzenberg, Hanan Amro, Joann I. Prisciandaro, Eduardo Acosta, Ian Gallagher, Don A. Roberts

**Affiliations:** ^1^ Department of Radiation Oncology University of Michigan Ann Arbor Michigan USA

**Keywords:** water equivalent, inhomogeneity, dose, density variation, phantom

## Abstract

The purpose of this study was to determine the dosimetric impact of density variations observed in water‐equivalent solid slabs. Measurements were performed using two 30 cm×30 cm water‐equivalent slabs, one being 4 cm think and the other 5 cm thick. The location and extent of density variations were determined by computed tomography (CT) scans. Additional imaging measurements were made with an amorphous silicon megavoltage portal imaging device and an ultrasound unit. Dosimetric measurements were conducted with a 2D ion chamber array, and a scanned diode in water. Additional measurements and calculations were made of small rectilinear void inhomogeneities formed with water‐equivalent slabs, using a 2D ion chamber array and the convolution superposition algorithm. Two general types of density variation features were observed on CT images: 1) regions of many centimeters across, but typically only a few millimeters thick, with electron densities a few percent lower than the bulk material, and 2) cylindrical regions roughly 0.2 cm in diameter and up to 20 cm long with electron densities up to 5% lower than the surrounding material. The density variations were not visible on kilovoltage, megavoltage or ultrasound images. The dosimetric impact of the density variations were not detectable to within 0.1% using the 2D ion chamber array or the scanning photon diode at distances 0.4 cm to 2 cm beyond the features. High‐resolution dosimetric calculations using the convolution–superposition algorithm with density corrections enabled on CT‐based datasets showed no discernable dosimetric impact. Calculations and measurements on simulated voids place the upper limit on possible dosimetric variations from observed density variations at much less than 0.6%. CT imaging of water‐equivalent slabs may reveal density variations which are otherwise unobserved with kV, MV, or ultrasound imaging. No dosimetric impact from these features was measureable with an ion chamber array or scanned photon diode. Consequently, they were determined to be acceptable for all clinical use.

PACS numbers: 87.55.km, 87.55.Qr

## I. INTRODUCTION

Water‐equivalent slabs are widely used in radiation therapy to perform both qualitative and quantitative quality assurance (QA) measurements. While AAPM Task Group 51 Report 67 requires the use of water for reference dosimetry measurements on an annual basis, it does not preclude the use of water‐equivalent materials for more frequent consistency checks and QA verification.^(1)^ Additionally, AAPM Task Group 106 allows the use of solid water‐equivalent materials for some commissioning measurements for convenience.^(2)^ The relative convenience and ease of use of solid slabs over water tanks makes the various water‐equivalent materials widely used. The properties of several of these materials have been previously reported for use in both photon and electron beams.^(^ ^3^ ^–^ ^11^ ^)^ Water‐equivalent (WE) materials are commonly available in slabs measuring $20\times 20\;{\text{cm}}^{2},30\times 30\;{\text{cm}}^{2}$ or $40\times 40\;{\text{cm}}^{2}$, with thicknesses ranging from 0.2 to 6.0 cm. They are commonly used in stacks which may be customized to place ion chambers or films at specific depths. Thus some slabs serve as buildup material while others provide backscatter. Since Solid Water 457 is frequently used to comply with TG‐106 and TG‐51 requirements and for verification of clinical techniques such as IMRT, a higher grade of material, Certified Therapy Grade, is available for situations which may impact dosimetric measurements. These slabs receive a higher level of QA verification by the manufacturer and come with additional data verifying some aspects of the quality of the material. In terms of homogeneity, this material is guaranteed not to contain air bubbles or other artifacts greater than 0.1 cm diameter within 5 cm of the center of the slab, and no more than two air bubbles or artifacts greater than 0.2 cm diameter in an annulus of 5 cm to 10 cm from the center. Measurements for purposes of making these assessments are made using film and 50 kVp X‐rays by an independent laboratory and included with the purchased material.^†^ Regardless of the quality of material purchased, occasionally a slab of WE material is observed to contain density variations when imaged with CT.^(12)^ While these defects do not appear on a diagnostic kilovoltage radiograph provided with therapy grade slabs, they are readily apparent on tomographic CT images. As observed in the commercially available pieces inspected here, in samples provided by Gammex, and according to personal communication with Gammex, these density variations may take the appearance of long cylindrical voids or as low‐density regions, perhaps due to the inclusion of micro‐bubbles or incomplete mixing of additives during the manufacturing process. The cylindrical density features are typically 0.1–0.3 cm in diameter and many centimeters long, while regions of low density may be many centimeters across. The striking nature of these density variations in CT images raises the question of their dosimetric impact on QA measurements, and the need for user‐specific QA of each piece of WE material that will be used for quantitative measurements.

In this study we examine the dosimetric impact of such density variations observed in Certified Therapy Grade Solid Water 457 water‐equivalent slabs. CT images were taken of samples of WE material containing density variations and used for dosimetric calculations to assess the impact of the inhomogeneities. Dosimetric calculations were also performed on simulated geometries with voids to understand the limits of sensitivity of calculations to small inhomogeneities. Additionally, measurements were performed using samples containing density variations, and are compared to the calculations.

## II. MATERIALS AND METHODS

The exact materials and technique for manufacturing Certified Therapy Grade (CTG) Solid Water 457 (Gammex/RMI, Middleton, WI) is proprietary information. However, the following details are known and available upon request from the manufacturer. This WE material is created from a mixture of solid and liquid components, which are stored in a temperature and humidity controlled environment. The components are combined in an industrial mixer under vacuum to reduce air bubbles that may otherwise be included or created during the mixing process. During the mixing process, the materials become very hot and the mixing time is carefully timed. Once mixing is complete, the material is gravity fed into molds through a hole in the bottom of the mixer while still under vacuum. When the mold is filled, it is removed from vacuum and placed in a temperature‐controlled chamber to harden. It is believed that the density variations described in this work form while the material hardens, and are reported by the manufacturer to be only observed in the thicker pieces. After the material has hardened, it is removed from the mold and cut to size and finished. The manufacturer then conducts quality assurance tests on each batch to determine if it meets the standards for commercial availability. Quality assurance tests to determine if a batch meets the criteria for Certified Therapy Grade are performed by an independent lab. The current tests performed on each batch of CTG include measures of ionization versus depth for 18 MeV electrons and 6 MV photons relative to water, a 50 kVp diagnostic radiograph to screen for air bubbles within 10 cm of the center of the slab, and verification of physical dimensions and mass density.

The relevant physical properties as provided by the manufacture are a measured physical density of $1.043\pm 0.005\;{g/\text{cm}}^{3}$, calculated electron density of ($0.562\pm 0.003)N^{A}\;{e/\text{cm}}^{3}$, and linear attenuation coefficient relative to water of 1.030±0.01 for photon energies from 100 kV to 2 MV. Dosimetric measurements were performed on two different slabs of $30\times 30\;{\text{cm}}^{2}$ Certified Therapy Grade Solid Water 457, 4 cm and 5 cm thick, which will be referred to as Sample A and B, respectively. Additional measurements were conducted on four sample pieces provided by the manufacturer, which failed pre‐commercial quality standards. Specific measurements and calculations designed to study the dosimetric impact beyond density variations are described below.

### A. Description of measurements

In the measurements described below, the extent of the inhomogeneities in the WE slabs was measured with CT imaging. Additional images were obtained with a megavoltage portal imaging device. Dosimetric measurements are made using a two‐dimension ion chamber array (MatriXX, Iba Dosimetry America, Inc., Bartlett, TN) and photon diode (Scanditronix Medical AB, model DEB010) scans within a Blue Phantom (Iba Dosimetry America, Inc., Bartlett, TN).

#### A.1 CT image studies

Upon the receipt of all new water‐equivalent slabs, initial quality screening is performed by a CT scan of each sample, per institutional policy. Scans were obtained with the samples placed horizontally (flat) and vertically on the CT couch at 120 kVp, 450 mAs, 1 mm thickness and in‐plane resolution of 0.098 cm×0.098 cm. Planar and cross‐sectional CT images showing the location and nature of the inhomogeneities in six water‐equivalent slabs were obtained. Density variations from images of commercially available Samples A and B were studied, and used for dose calculations and measurements. To identify the orientation of the slabs, fiducial line markers were placed on the edge of the slabs, which will be referred to as the superior edge. In addition, regions with density variations were placed toward the bottom (inferior) when the slabs were placed horizontally. This orientation was chosen to place the density variations as close as possible to detectors placed under the slabs.

#### A.2 Megavoltage portal imaging

**Figure 1:**
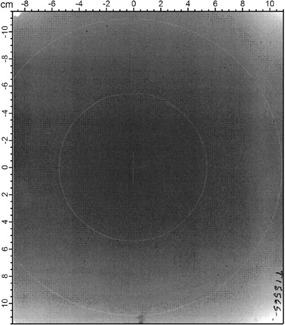
The diagnostic film which was provided with Certified Therapy Grade Solid Water 457, Sample A, demonstrating the absence of voids within a 5 cm and 10 cm radius of the center of the slab.

When Certified Therapy Grade Solid Water is purchased, Gammex/RMI provides a kilovoltage radiograph film of each slab, obtained as part of the Therapy Grade certification process. This serves to demonstrate that there are no observable voids greater than 0.1 cm in diameter or defects as measured by kV imaging within the central 5 cm radius, and no more than two within the central 10 cm radius. Figure 1 shows a digitized image of the film provided with Sample A. No voids or defects are visible.

Radiographic measurements were also made using a 6 MV treatment beam where the samples were placed directly on top of an amorphous silicon flat panel imager with 784 micron pixel pitch (IAS2, Varian Medical System, Palo Alto, CA). Areas known to contain density variations, as observed in CT images, were imaged.

#### A.3 Ultrasound imaging

In an attempt to determine whether voids or defects in the WE slabs could be realized with other imaging modalities – possibly a device that would be more cost effective – the use of ultrasound imaging was explored. In turn, Samples A and B were placed horizontally in a large water tank. The tank was filled to allow 1–1.5 cm of liquid water to cover the surface of the WE slabs. The areas of interest in Samples A and B were then scanned using the Siemens ACUSON Antares (Siemens Medical Solutions, Malvern, PA) with the CH4‐1, CH6‐2, and VFX13‐5 transducers. The CH4‐1 and CH6‐2 are convex transducers with frequency ranges of 4–1 MHz and 6–2 MHz, respectively, and the VFX13‐5 is a linear transducer with a frequency range of 13–5 MHz.

#### A.4 Ion chamber array measurements

Relative dosimetric measurements were made with a 2D ion chamber array. The array was first calibrated relative to the machine output as follows. The array was powered up at least 30 minutes before beginning measurements and pre‐irradiated with 10 Gy to minimize warm‐up effects, as recommended by the manufacturer. The array was aligned to isocenter, with 5 cm of water equivalent material placed above and below the device and set to an SSD of 95 cm. (These slabs of material were previously determined to be of uniform density by CT imaging.) Using a field size of 10 cm×10 cm,100 MU was delivered and the measured dose defined, for purposes of calibration, to be 94.6 cGy, based on TPR and inverse square law corrections from treatment calibration conditions.

##### A.4.1 Measurement of sample material defects

**Figure 2:**
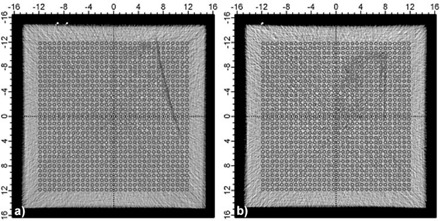
CT images of: a) Sample A and b) Sample B, showing representative density variations relative to the positions of the individual ion chambers in the 2D array.

To measure the relative dosimetric effect of inhomogeneities within each slab of material, each slab was used as its own control to avoid possible complications of density variations relative to other slabs. This was accomplished by placing a slab on top of the array and performing two measurements. The first was performed at a known arbitrary orientation, as shown in Fig. 2, while the second measurement was performed with the slab rotated 180° about the vertical axis. The defects were placed so that they were as close to the surface of the ion chamber array as possible. In Sample A, the density variations range from 0.7–2.2 cm from the bottom surface of the array, while in Sample B they are 0.8–1.9 cm from the bottom surface. The first measurement was subtracted from the second and divided by the average of the two measurements to show relative variation. The average and standard deviation of the relative differences among the ion chambers under the density variations in each orientation were found and compared.

##### A.4.2 Measurement of simulated geometrical defects

In order to measure the sensitivity of the 2D ion chamber array to long voids of varying spatial extent, geometrical defects were simulated with thin water‐equivalent slabs and measured. In the first measurement, $M_{u}$, a uniform reference field was obtained by placing a 0.2 mm WE slab on the array, followed by a 5 cm thick slab. A uniform 28 cm×28 cm field was delivered with 200 MU for this and all subsequent measurements in this series. In the next measurement, $M_{g=0}$, a second 0.2 cm thick slab was abutted (i.e., gap, g=0) with the first so their adjoining edges were centered over a column of ion chambers in the radial beam direction. In the next five measurements, $M_{g=1-5}$, the abutting edges of the 0.2 cm slabs were separated in increasing 0.10 cm intervals and the gap centered over the same row of ion chambers. Over the course of the measurements, the response of the array increased slightly (< 0.5%) as the electronics warmed up, despite using a warm‐up procedure surpassing that recommended by the manufacturer by at least 15 minutes. It is possible that the increase was partially due to drifts in temperature and pressure, though the increase was consistently observed on multiple refinements of the measurements. This effect was distinct in that it influenced all chambers, while the experimental changes only affected a single row of detectors. This effect was removed by scaling the average response of each $M_{g}$ to that of $M_{u}$ over 6×6 chambers to the right of the center of the array (away from the simulated gap), so that $M_{g}’=M_{g}(M_{u}/M_{g})6\times 6$. The relative difference compared to the initial measurement was determined as $D_{g}=(M_{g}’-M_{u})/M_{u}$. The columns of ion chambers were then averaged to find the average percent difference relative to the initial open field.

#### A.5 Water tank scans with photon diode (with and without SW)

To avoid the volume averaging implicit to the ion chambers of the previously‐described measurements, additional measurements were made with a photon diode. Slab A was suspended in a Blue Phantom just under the surface of the water using a four‐pound test monofilament line. The photon diode was scanned through water approximately 1 cm under the slab, across its width at 2 cm intervals. The slab was irradiated with a 6 MV uniform 34 cm×34 cm field at 600 MU/min and a diode speed of 1.5 cm/s. Data from the photon diode were normalized to a reference chamber placed above the phantom in a corner of the field.

### B. Description of dose calculations

Dosimetric calculations were performed on the simulated slab geometries to validate the correspondence with measurements. Calculations were also performed based on the CT scans of Samples A and B.

#### B.1 Sample water equivalent slabs with inhomogeneities

Dose calculations were performed using the convolution–superposition algorithm in the in‐house treatment planning system, UMPlan. A single 6 MV, 10 cm×10 cm beam was incident normal to the slab surface in each calculation. In the first calculation, the beam traversed the thinnest dimension of the slab which also traversed the shortest span of density variation. In the second calculation, the beam was parallel to the long dimension of the slab, and traversed the longest span of density inhomogeneity. The convolution–superposition (CVSP) algorithm was used to calculate dose on uniform 3D voxels of 0.08 cm per side. Hounsfield units of the CT scan were converted to electron density at the native 0.1 cm×0.1 cm×0.1 cm resolution of the original scan.

#### B.2 Simulated geometries

To validate the sensitivity of the calculations compared to measured results, the doses to the ion chambers below the simulated tubular voids were calculated using CVSP. To include the volume averaging effect of the ion chambers, 84 dose calculation voxels of volume 0.1 cm^3^ on a uniform 3D grid, were placed in and around each cylindrical ion chamber of the 2D array. The average dose to each set of 84 voxels was taken to be the calculated dose for each ion chamber for comparison to measurements. (This technique has been in clinical use for IMRT QA for three years at our institution.)

## III. RESULTS & DISCUSSION

### A.1 CT image studies

**Figure 3:**
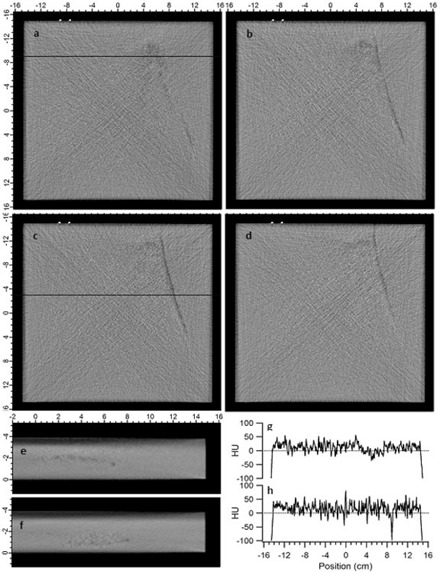
CT images of Sample A showing representative density variations (a)–(d) in the plane of the slab, and in the cross section (e) and (f) at the location indicated by the horizontal lines in (a) and (c), respectively. Line profiles (g) and (h) are shown from (a) and (c), respectively, as indicated by the horizontal lines.

**Figure 4:**
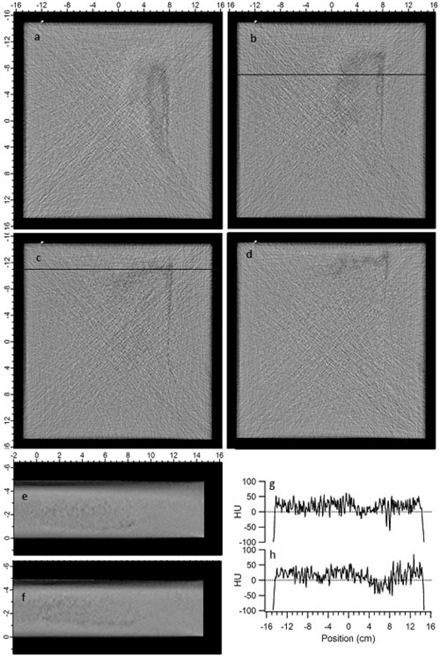
CT images of Sample B showing representative density variations (a)–(d) in the plane of the slab, and in the cross section (e) and (f) at the location indicated by the horizontal lines in (b) and (c), respectively. Line profiles (g) and (h) are shown from (b) and (c), respectively, as indicated by the horizontal lines.

The density variations observed in Samples A and B are shown in Figs. 3 and 4, respectively. All CT image values are displayed in Hounsfeld units (HU) with a display window of ± 250 HU centered at a level of 0 HU. In Sample A, density variations were observed on 15 consecutive 0.1 cm thick slices ranging from positions 0.7–2.2 cm from the bottom surface. (Figs. 3a)– 3(d) show four representative images in the plane of the water equivalent slab at 1.0 to 1.3 cm, respectively, from the bottom of the slab. (Figure 3e)and (3f) show cross‐sectional images of Sample A taken 6.0 cm and 12.0 cm, respectively, from the superior end, as indicated by the horizontal line in (Figs. 3a)and (3c). (Figures 3g)and (3h) show line profiles through (Figs. 3a)and (3c), as indicated by the horizontal lines. In Sample B, density variations were observed on 12 consecutive 0.1 mm thick slices ranging from 0.8–1.9 cm from the bottom surface. (Figures 4a)–4(d) show four representative images in the plane of the water‐equivalent slab at 0.9, 1.1, 1.3 and 1.5 cm, respectively, from the bottom of the slab. (Figures 4e)and (4f) show cross‐sectional images of Sample B taken 6.0 cm and 4.0 cm, respectively, from the superior end, as indicated by the horizontal line in (Figs. 4b)and (4c). (Figures 4g)and (4h) show line profiles through (Figs. 4b)and (4c), as indicated by the horizontal lines.

In both samples, the density variations are located in the superior right side and posterior (bottom) half of the slab, and extend from the superior edge inferiorly for at least 20 cm. In the samples shown here, two distinct features are observed in the density variations: large regions of slightly lower than average HU values, and long thin “worm holes” of lower HU value. The larger regions have a maximum extent up to 8 cm wide, 15 cm long and 2 cm thick, though typically smaller and thinner within this bounding volume. The low density worm holes are typically 0.2 cm in diameter and up to 20 cm long.

The average and standard deviation of values in uniform regions in Sample A and B are $16.2\pm 25.3(\sigma_{\text{mean}}=0.42)$ HU and $18.7\pm 26.1(\sigma_{\text{mean}}=0.44)$ HU, respectively. In the large regions of density variation the values are $-10.9\pm 26.9(\sigma_{\text{mean}}=0.89)$ HU and $4.3\pm 28.0(\sigma_{\text{mean}}=0.61)$ HU, in Samples A and B, respectively. In the long cylindrical low‐density regions, average values were $-34.2\pm 34.9(\sigma_{\text{mean}}=1.5)$ HU and $-15.7\pm 32.3(\sigma_{\text{mean}}=1.9)$ HU, in Samples A and B, respectively. Thus, in Sample A, the large regions of variation are on average 27 HU lower than the uniform regions, or 2.7% lower electron density, while in Sample B, it is 14 HU or 1.4% lower electron density. In the long cylindrical regions, the average value in Sample A is 50 HU lower, or 5% lower electron density, while in Sample B, it is 34 HU, or 3.4% lower electron density. While the appearance and use of the term “worm hole” gives the impression of a physical void within the material, this is not the case. Scrap pieces were imaged with CT to determine the location of such features. Then a milling machine was used to remove the overlying material. No physical voids were observed, with typically little to no visible difference from the surrounding material. In one scrap sample, density variations in the CT were found to coincide with thin interior regions with slightly darker and lighter color. The features are solid material that is simply a few percent less dense. Because CT images are displayed with windowing and leveling automatically determined based on the range of values in the scanned object, small variations in an otherwise uniform object may appear black, like air, giving the visual impression of a worm hole.

### A.2 Megavoltage portal imaging

**Figure 5:**
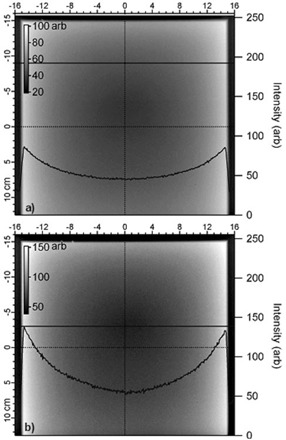
Megavoltage images of: (a) Sample A (4 cm) and (b) Sample B (5 cm), respectively. Line profiles are shown at the position of the horizontal solid lines. As in diagnostic X‐ray images, no density variations are visible.

Megavoltage portal images of Samples A and B are shown in Fig. 5. Line profiles are superimposed on the images which were obtained from the positions indicated by the horizontal lines. Line profiles from the same positions in the CT scans are also shown in Figs. 3 and 4. Despite placing the WE slabs directly on top of the EPID housing to reduce magnification of source penumbra, no density variations are distinguishable from signal noise. The “cupped” nature of the profile is due to the divergent beam geometry traversing greater thickness of material at increasing angles from central axis. This result is largely expected due to the low signal present in the latent image, the size of the source, and the noise in the image.

### A.3 Ultrasound imaging

Ultrasound imaging is dependent on the interactions of acoustic waves within medium. At interfaces, the incident wave can be absorbed, reflected, refracted, and/or scattered. To obtain an ultrasound image, echoes from the initial megahertz pulse must be detected by the transducer. While higher frequencies with shorter wavelengths provide higher resolution, lower frequencies have lower absorption coefficients and are able to image density variations at greater depths. A range of frequencies from 1 to 14 MHz were used in these tests.

Three ultrasound transducers were tested on Samples A and B to determine whether the voids or defects identified by CT imaging were visible. The first probe tested was the VFX13‐5. This flat, linear probe had a nice, smooth contact with the surface of the WE slabs. The probe was used to scan the areas of interest for both Samples A and B. However, even at its lowest frequency which gives the highest penetration, the voids were not visible. In the hope that these artifacts might be apparent using a lower‐frequency probe, the CH6‐2 and CH4‐1 probes were also tested. Both transducers have a convex face, making it more challenging to achieve a good contact with the flat, solid surface of the WE slabs, though the effect of this difficulty was minimized by using liquid water to improve mechanical contact. The frequency was set to the lowest achievable for both probes, and the surface of the slabs in the regions of interest were scanned. Unfortunately, due to the convex shape of the transducers, the echoes received by the lateral‐most elements created a ring pattern that resulted in a vacant dark spot at the center of the image. Similar to the VFX13‐5, the voids in WE slabs were not visible.

While the shape of the transducer may have obscured the image, we believe a lower‐frequency linear probe would also not have been effective. Although the physical density of the WE slabs are consistent with liquid water $(1.043\pm 0.005\;{g/\text{cm}}^{3}$ versus $1.0\;{g/\text{cm}}^{3}$), large differences in the acoustic impedance (Z =ρ v) of these media are indicative of significant differences in their speed of sound. Measurements of the speed of sound in the WE slabs were not found in literature; however, we hypothesize that it may be comparable to ice, 3.94 mm/μs.^(13)^ Imaging through or within solid materials presents special challenges. The speed of sound in solids is several times higher than in liquids making the acoustic impedance proportionally higher also. As well, the absorption of acoustic energy, which is generally dependent on frequency, viscosity and the relaxation time of the material, may be very high in solids compared to water. For example, the absorption coefficient of water at 1 MHz is 0.0022 dB/cm, while in aluminum and bone it is 0.018 dB/cm and 20 dB/cm, respectively. Consequently, without specialized equipment, it may be very difficult to image density variations in solid materials.

### A.4 Ion chamber array measurements

Measurement results using a 2D ion chamber array to investigate the impact of density variation in sample slabs of water‐equivalent material and in simulated inhomogeneities are discussed in the sections below.

#### A.4.1 2D ion chamber array measurements on sample materials

**Figure 6:**
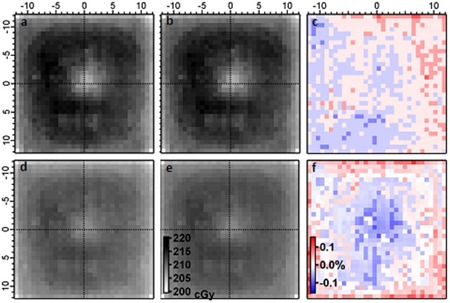
Ion chamber array measurements for Sample A at: (a) zero degree and (b) 180° orientations, and for Sample B in (d) and (e), respectively. The range in dose levels for (a), (b), and (d) are consistent with those displayed in (e). The images are subtracted from each other and divided by the average of the two images for: (c) Sample A and (f) Sample B. The range of the color scale shown in (f) is also used in (c).

In the analysis performed on these measurements, measurable dosimetric deviations caused by the density variations would produce rotationally symmetric hot and cold spots in the 2D image. The results for Sample A are shown in (Figs. 6a)–6(c), while those for Sample B are shown in (Figs. 6d)–6(f), respectively. In (Figs. 6b)and (6e), the respective samples have been rotated 180°, while (Figs. 6c)and (6f) show the differences between the images for the respective samples. In Fig. 6(c), the upper right portion of the image appears slightly hot, while the lower left appears slightly cold. However, distinct symmetrical signals corresponding to the positions of the density variations are not apparent in either difference image. Any signals caused by density variations in the WE slabs are below the 0.1% level.

#### A.4.2 Measurement of simulated geometrical defects

**Figure 7:**
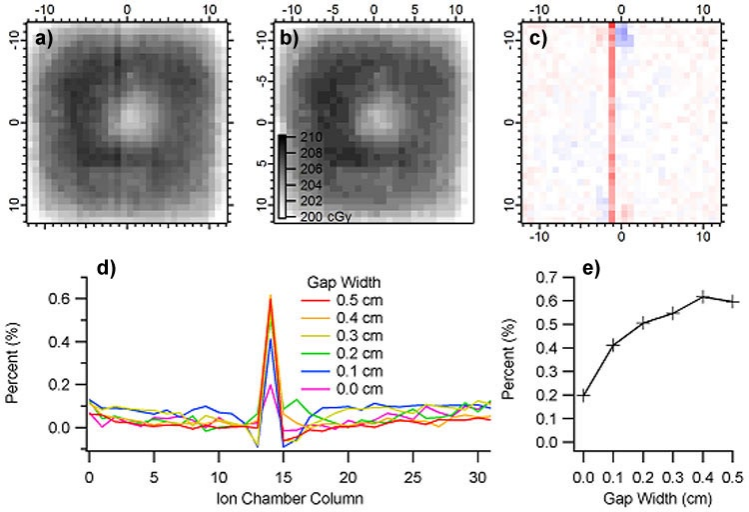
Ion chamber array measurements of: an 0.5 cm wide by 0.2 cm high by 30 cm long (a) void formed with WE slabs, and a uniform phantom (b) of the same thickness. The percent difference of (a) and (b) is shown in (c). The average column value of the percent difference in (c) is shown in (d), along with all results for gaps of 0.0–0.5 cm. The percentage increase in dosimetric response versus gap width is shown in (e).

Figure 7 shows the measurement of the 0.5 cm wide by 0.2 cm high gap created between two, 0.2 cm thick, WE slabs, which were placed under a 5 cm slab. The reference uniform measurement and the percent difference images are also shown for comparison. The average column value of the percent differences relative to the uniform measurement is shown for gaps ranging from 0.0 to 0.5 cm wide. The average percent increase in dose versus gap width starts at +0.2% for zero gap and increases to 0.6% for a 0.5 cm wide gap, which is slightly wider than the 0.45 cm diameter of the ion chamber.

### A.5 Water tank scans with a photon diode

**Figure 8:**
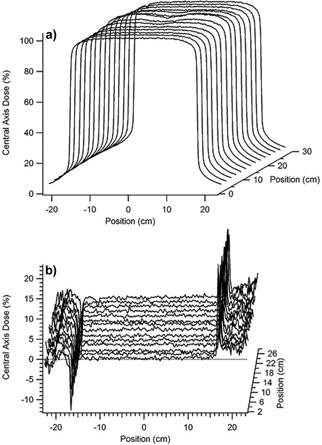
Cross‐plane profiles (a) measured with a photon diode scanned roughly 1 cm under Sample A which was suspended just under the surface of the water in a water tank. The percent difference from scans in water, without Sample A in place, are shown in (b). The average difference between the scans, with and without Sample A in place, was 0.0%± 0.3%.

**Figure 9:**
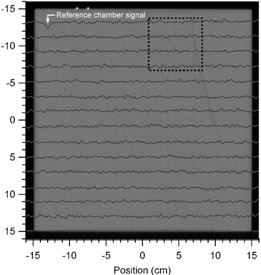
The percent difference from photon diode cross‐plane profile scans in water, with and without Sample A suspended just below the water surface (100 cm SSD, depth of 6 cm). The large deviation in the upper left corner is due to the reference chamber. Average percent differences are similar when measured under the whole slab (0.0%± 0.3%) and in the dotted region outlining known density variations (0.1%± 0.2%).

Cross‐plane scans (100 cm SSD) obtained with a photon diode scanned at a depth of 6 cm, roughly 1 cm below Sample A, in a water tank are shown in Fig. 8(a). Similar scans were taken without the WE slabs in the water, with the same SSD and depth. The percent difference between the scans is shown in Fig. 8(b). These differences were overlaid on a CT of Sample A showing the relative position of the density variations (see Fig. 9). The largest difference of −2.5%, seen in the upper left corner, is due to the placement of the reference ion chamber. Omitting measurements under the reference chamber, the average percent difference between the scans with WE material and water alone is 0.0±0.3%(range=[−1.1,1.2]%). In a 6 cm×6 cm region under the density variations (shown by the dotted square in Fig. 9), the average percent difference is −0.1±0.2% (range = [−0.9, 0.6]%). Also, by visual inspection, there is no apparent change in the relative dose in the regions known to have density variations, as observed in the CT images in Fig. 9.

## B. Calculations and simulations

In Section B.1 below, the results of the CVSP calculations, with density corrections enabled, on the CT scans of Sample B are shown. Similar calculations on simulated inhomogeneities are shown in Section B.2 below.

### B.1 Dose calculations on CT scans

**Figure 10:**
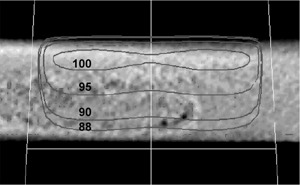
Isodose lines at 100%, 95%, 90% and 88% dose, for 10 cm×10 cm beam incident on Sample B. Isodose lines, which are just below the density variations, show no impact from the inhomogeneity.

The CVSP calculation results of a 10 cm×10 cm field normal to the large surface of Sample B are shown in Fig. 10. Isodose lines are shown for 100%, 95%, 90% and 88% doses relative to the maximum dose. The 90% and 88% isodose lines pass just below the cylindrical density variations, located just to the right of isocenter at a depth of about 4.5 cm. The density variations are seen to have no impact on the isodose line just below the inhomogeneity.

**Figure 11:**
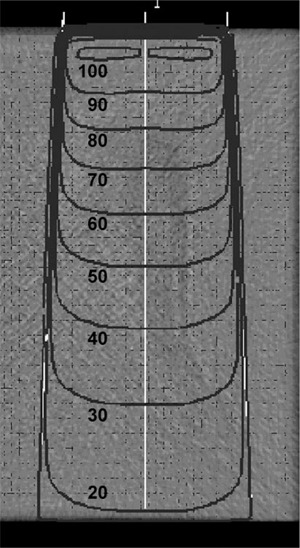
Isodose lines for a 10 cm×10 cm beam, which are just below the density variations, show no impact from the density variations after passing through at least 10 cm of the low‐density region.

The CVSP calculation results when a 10 cm×10 cm beam traverses the density variations through the long dimension of Sample B are shown in Fig. 11. Despite the long path length through the region with density variations parallel to the beam axis, there is no impact on the dose distribution.

### B.2 Calculations on simulated geometries

**Figure 12:**
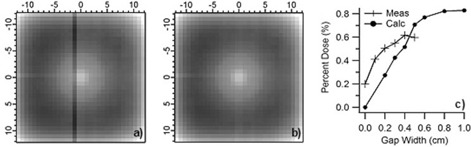
Calculation of the dose to each ion chamber in a 2D array with (a) a 0.5 cm wide by 0.2 cm high air gap centered 0.3 cm above a column of chambers, and (b) no air gap and with 5 cm of water equivalent material on top. The average dose to each ion chamber in the column versus gap width is shown in (c) (N.B. calculations as dots, measurements as crosses).

The results of the dosimetric calculations on the simulated gaps are shown in Fig. 12. In Fig. 12(a), the results of the 0.2 cm high×0.5 cm wide air gap centered over a column of ion chambers are shown, while in Fig. 12(b) the calculation with no gap is shown. (Figure 12c) shows the average percent increase in dose due to the gap over the column of ion chambers for the calculations and the measurements. Note that while the calculations increase linearly with gap width up to 0.5 cm, the measurements increase rapidly with small gaps and show a smaller increase in dose with larger gaps. This behavior is expected based on the measurement and calculation geometries. The dose to the ion chamber is calculated on a cubic uniform 3D grid of 0.1 cm voxels centered in and around the chamber. As the gap width increases, a linearly increasing number of calculation voxels is exposed. In the measurement geometry, the projection of the rectangular gap bisects the cylindrical ion chamber. As the gap width increases, the increase in the proportion of the cylindrical volume under the gap diminishes. The measured and calculated doses converge, as the gap width approaches the width of the ion chamber. The calculations increase another 0.1% for gap widths of 0.8 cm and 1.0 cm. The calculation and measurement agree within 0.1%–0.2% for a 0.5 cm wide gap centered over the 0.45 cm diameter ion chambers.

These results on simulated void geometries serve to demonstrate the sensitivity of the calculations and measurements to the presence of density variations in the water equivalent slabs. While the simulated inhomogeneities studied here have an assigned electron density of zero, the electron density of the dose variations in Samples A and B, relative to water, is at least 95% or greater. Consequently, the dosimetric impact of the roughly 0.2 cm diameter cylindrical density variations observed in Samples A and B is expected to be far less than the 0.5% dose increase measured due to a 0.2 cm×0.2 cm void. Based on these result, the impact of the observed density variations is not expected to have a detectable dosimetric impact on measurements.

## IV. CONCLUSIONS

CT imaging of water‐equivalent slabs may reveal density variations that are otherwise unobserved with kV, MV, or ultrasound imaging. Two general types of density variation features were observed and reported by the manufacturer: 1) regions up many centimeters across, but typically only a few millimeters thick, with electron densities a few percent lower than the bulk material, and 2) cylindrical regions roughly 0.2 cm in diameter and up to 20 cm long, with electron densities up to 5% lower than the surrounding material. Physical inspection of samples, machined to expose density variations located by CT imaging, revealed solid material at these locations with little to no visible difference from the surrounding material. The density variations were not visible on kilovoltage or megavoltage images. The dosimetric impact of the density variations were not detectable to within 0.1% using the 2D ion chamber array or the scanning photon diode at distances 0.4 cm to 2 cm beyond the features. High‐resolution dosimetric calculations using the convolution–superposition algorithm with density corrections enabled on CT‐based datasets showed no discernable dosimetric impact. Calculations and measurements on simulated voids measuring 30 cm long by 0.2 cm high and ranging from 0 to 1.0 cm wide place the upper limit on possible dosimetric variations from observed density variations at much less than 0.6%.

## ACKNOWLEDGMENTS

We would like to acknowledge and thank Gammex/RMI for graciously exchanging pieces of Solid Water, providing scrap pieces for examination, and for helpful discussions and information on the manufacturing process and product quality control procedures and standards.
